# The Medical News Visiting List for 1900

**Published:** 1899-12

**Authors:** 


THE MEDICAL NEWS VISITING LIST FOR 1900. Weekly (dated, for 30 patients); monthly (undated, for 120 patients per month); perpetual (undated, for 30 patients weekly per year); and perpetual (undated, for 60 patients weekly per year). The first three styles contain 32 pages of data and 160 pages of blanks. The 60-patient perpetual consists of 256 pages of blanks. Each style in one wallet-shaped book, with pocket pencil and rubber. Seal Grain Leather, $1.25. Thumb-letter Index, 25 cents extra. Philadelphia and New York: Lea Brothers & Co.

A visiting list is an indispensable convenience for the active practitioner. Its carefully adapted blanks enable him at once to note clinical details or every day work, as well as charges and receipts, and to unburden his memory from what can better be carried on paper. It also furnishes him with a legal record necessary for the collection of delinquent bills. Prominent among the many books of this nature stands the Medical News Visiting List. Its blank pages are arranged to classify and record memoranda and engagements of every description occurring in the practice of the physician, surgeon or obstetrician. The work opens with thirty-two pages of printed data of the most useful sort, including an alphabetical Table of Diseases with Approved Remedies, a Table of Doses, Sections on Examination of Urine, Artificial Respiration, Incompatibles, Poisons and Antidotes, a Diagnostic Table of Eruptive Fevers, and a full page plate showing at a glance the incisions for ligation of the various arteries, an invaluable guide in such emergencies. The Medical News Visiting List is issued in four styles, adapted to any system of records and any method of keeping professional accounts. It is printed on fine, tough paper, suitable for pen or pencil, and durably and handsomely bound in the size of a wallet for the pocket. When desired, a Ready Reference Thumb-letter Index is furnished, which is an economizer of time.

